# RETRACTED: Wu et al. Silencing of *Kv1.5* Gene Inhibits Proliferation and Induces Apoptosis of Osteosarcoma Cells. *Int. J. Mol. Sci.* 2015, *16*, 26914–26926

**DOI:** 10.3390/ijms262110549

**Published:** 2025-10-30

**Authors:** Jin Wu, Zhida Chen, Qingjun Liu, Wenrong Zeng, Xinyu Wu, Bin Lin

**Affiliations:** 1 Department of Orthopaedics, The Affiliated Southeast Hospital of Xiamen University, Orthopaedic Center of People’s Liberation Army, Zhangzhou 363000, China; 2 Department of Neurology, The Affiliated Southeast Hospital of Xiamen University, Zhangzhou 363000, China

The journal retracts the article “Silencing of *Kv1.5* Gene Inhibits Proliferation and Induces Apoptosis of Osteosarcoma Cells” [1] cited above.

Following publication, concerns were brought to the attention of the Editorial Office regarding the integrity of a number of images presented in this publication [1].

Adhering to our standard procedure, an investigation was conducted by the Editorial Office and Editorial Board that identified indications of inappropriate editing and partial duplication between figures presented in this article [1]. As the authors remained unresponsive, the Editorial Board has lost confidence in the reliability of the findings and has decided to retract this publication, as per MDPI’s retraction policy (https://www.mdpi.com/ethics#_bookmark30, accessed on 22 September 2025).

This retraction was approved by the Editor-in-Chief of the *International Journal of Molecular Sciences*.

The authors did not comment on this decision.
